# Curcumol inhibits malignant biological behaviors and TMZ-resistance in glioma cells by inhibiting long noncoding RNA FOXD2-As1-promoted EZH2 activation

**DOI:** 10.18632/aging.203662

**Published:** 2021-11-05

**Authors:** Xuyang Lv, Jiangchuan Sun, Linfeng Hu, Ying Qian, Chunlei Fan, Nan Tian

**Affiliations:** 1 Molecular Medicine Institute, Life Science College, Zhejiang Chinese Medical University, Hangzhou, Zhejiang, China

**Keywords:** curcumol, lncRNAs, epigenetic modification, EZH2, glioma

## Abstract

Currently, conventional treatment is not sufficient to improve the survival of glioma patients. Hence, adopting novel personalized treatment programs is imperative. Curcumol, a Chinese herbal medicine extract from the roots of *Rhizoma Curcumae*, has attracted significant interest due to its beneficial pharmacological activities. The current study revealed that curcumol inhibited the proliferation, metastasis, self-renewal ability, and TMZ resistance in glioma cells *in vitro* and *in vivo*. Next, the potential molecular mechanisms of curcumol in inhibiting glioma were investigated. We found that the long non-coding RNA (lncRNA) FOXD2-As1 might contribute to the effects of curcumol on glioma cells. Enforced expression of FOXD2-As1 attenuated the curcumol-induced reduction in glioma cell proliferation, metastasis, self-renewal ability, and TMZ resistance. Moreover, the forced expression of FOXD2-As1 reversed the inhibitory effect of curcumol on the binding ability of EZH2 and H3K27me3 modification in the promoter regions of anti-oncogenes. Our results showed for the first time that curcumol is effective in inhibiting malignant biological behaviors and TMZ-resistance of glioma cells by suppressing FOXD2-As1-mediated EZH2 activation. Our study offers the possibility of exploiting curcumol as a promising therapeutic agent for glioma treatment and may provide an option for the clinical application of this natural herbal medicine.

## INTRODUCTION

Traditional Chinese medicine (TCM) has been practiced for more than thousand years [1]. It is widely accepted today, and its popularity is growing internationally, including in Japan and the UK [2, 3]. Herbal medicine is the most important part of TCM, as it provides curative treatments for a number of diseases and physiological conditions [4–7]. Curcumol, a sesquiterpenoid extract from the herb *Rhizoma Curcumae*, has attracted significant interest recently owing to its beneficial pharmacological activities. It exhibits numerous therapeutic effects, such as anti-fibrotic, anti-oxidant, anti-inflammatory, and antic-ancer activities with low cytotoxicity [8, 9]. In recent times, several researches revealed that curcumol inhibits the growth of various types of cancer cells, including gastric adenocarcinoma, melanoma, hepatic cancer, and colorectal cancer cells [10–13]. Moreover, curcumol is lipophilic and readily crosses the blood–brain barrier [14, 15]. Therefore, we aimed to explore whether curcumol has inhibitory effects on glioma.

Glioma is the most commonly observed brain malignancy and is identified by several somatic mutations and aberrant activation of inflammatory responses [16]. The standard treatment for glioma patients comprises postoperative radiotherapy and adjuvant temozolomide (TMZ)-based chemotherapy [17]. However, therapeutic resistance and tumor recurrence seriously affect their efficacy. Thus, development of new therapeutic strategies against glioma is important.

Enhancer of zeste homolog 2 (EZH2) is a critical subunit of polycomb repressive complex 2 (PRC2). PRC2 causes the methylation of histone 3 lysine 27(H3K27me3) and closes the chromosome to epigenetically silence gene expression [18]. EZH2 plays a tumorigenic role by epigenetically activating oncogenic signaling pathways [19]. Several studies have shown that inhibition EZH2 expression in tumor cells can suppress the proliferation, migrative and invasive ability, and angiogenesis while increase the apoptotic rate [20–22]. In glioma, EZH2 was declared to be overexpressed in tumor tissues, and elevated EZH2 expression is closed related to the glioma grade and a bad prognosis [23, 24]. EZH2 has an important impact on stem-like cell maintenance of glioma [25]. Recent studies have suggested that the lncRNA FOXD2-As1 recruits EZH2 to reduce the expression of H3K27me3 in the promoter regions of anti-oncogenes to epigenetically regulate the malignant progression of tumors [26–28]. FOXD2-As1 is overexpressed in glioma and acts as an oncogene that promotes glioma malignancy and tumorigenesis [29, 30]. Our previous results showed that curcumol treatment markedly reduced FOXD2-As1 expression in glioma cells. Therefore, we studied on whether curcumol inhibited the development of glioma by suppressing FOXD2-AS1-mediated, EZH2-induced H3K27me3 expression in the promoter regions of anti-oncogenes. In this study, we tested this hypothesis to clarify the characteristics and molecular mechanisms underlying the effect of curcumol treatment on glioma cells.

## RESULTS

### Curcumol inhibited proliferation and metastasis and promoted apoptosis in glioma cells

**Figure 1:**
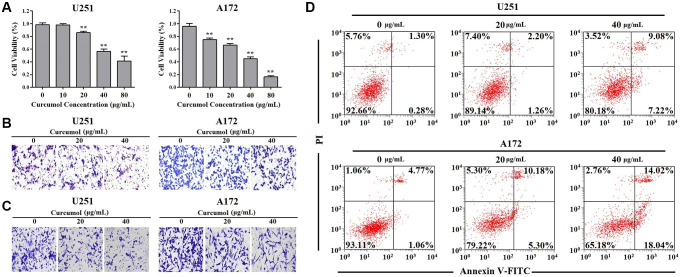
**Curcumol inhibited the proliferation and metastasis and promoted apoptosis in glioma cells.** (**A**) MTT assay was performed to determine the effect of curcumol on the proliferation of glioma cells. (**B**) Transwell migration and (**C**) invasion assays were performed to determine the effect of curcumol on the metastasis of glioma cells. (**D**) Glioma cells were treated with curcumol for 48 h and analyzed by flow cytometry after Annexin V-FITC/PI staining. Data were represented as means ± SD from at least of three independent experiments. ^*^p < 0.05, ^**^p < 0.01 when compared with the untreated control group.

To investigate the function of curcumol in glioma, we first detect whether curcumol had effect on glioma cells proliferation. MTT assay results showed that curcumol significantly decreased the viability of U251 and A172 cells, except at a dose of 10 μg/mL (Figure 1A). Curcumol displayed a dose-dependent cytotoxic effect against A172 cells (Figure 1A). Curcumol was also found to inhibit the migrative and invasive abilities of these two glioma cell lines, as shown in Figure 1B and 1C, wherein cellular treated with 20 and 40 μg/mL curcumol clearly inhibited cell migration and invasion. Further, to verify whether the inhibitory effect of curcumol on glioma cells is related to cell apoptosis, we determined the apoptosis-inducing effect of curcumol in U251 and A172 cells. The results of Annexin V-FITC/PI staining analysis showed that curcumol induced glioma cell apoptosis in a dose-dependent manner at 48 h after treatment (Figure 1D).

### Curcumol inhibited the self-renewal ability of glioma cells

**Figure 2:**
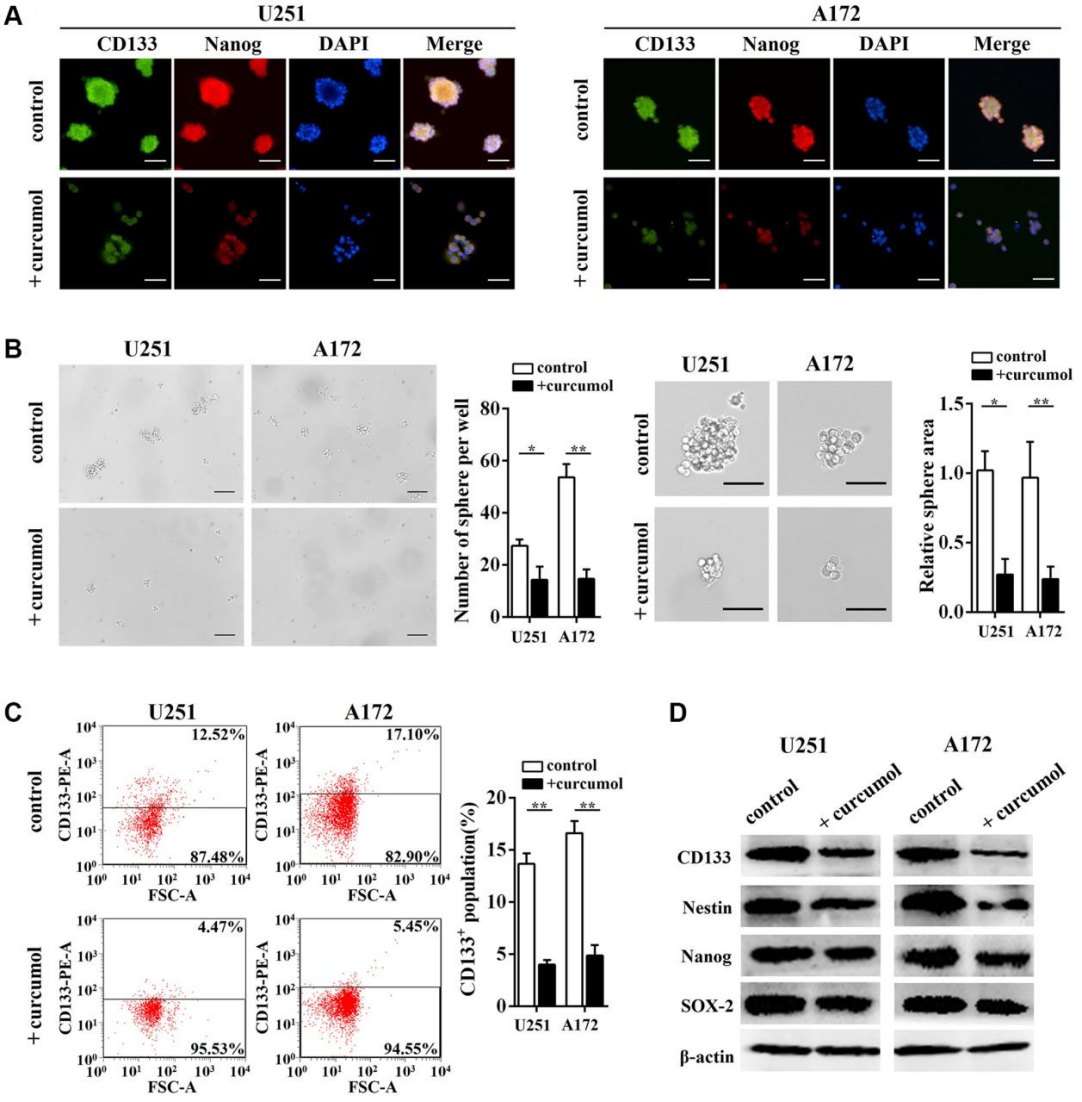
**Curcumol inhibited the self-renewal ability of glioma cells.** (**A**) CD133, Nanog, and DAPI staining were shown by immunostaining in neurosphere from glioma stem-like cells with or without 40 μg/mL curcumol. Scale bar = 200 μm. (**B**) Neurosphere formation capacity of glioma stem-like cells was determined. Curcumol treatment reduced the neurosphere formation of glioma stem-like cells, Scale bar = 150 μm. (**C**) The percentage of CD133+ cells was analyzed by FCM. (**D**) The expressions of stem cell marker genes in glioma cells were detected by western blotting analysis. ^*^p < 0.05, ^**^p < 0.01 when compared with the untreated control group.

As shown in Figure 2A, we enriched CD133+ “stem-like” neurospheres from U251 and A172 cells by culturing them under stem-like conditions. The impact of curcumol on the self-renewal ability of glioma cells was determined by using neurosphere formation analysis and the data indicated that curcumol (40 μg/mL) reduced the number of spheres compared to the control group and curcumol-treated spheres were markedly smaller than the control spheres (Figure 2B). Immunofluorescence results showed that curcumol treatment significantly decreased CD133 and Nanog protein levels when the glioma cell lines were cultured as neurospheres (Figure 2A). To estimate the percentage of CD133+ “stem-like” cells in the cancer cell population after curcumol treatment, the expression of CD133 was evaluated using flow cytometric analysis (FCM). Our data demonstrated that curcumol treatment reduced the percentage of CD133+ cells in both U251 and A172 cells (Figure 2C). In addition, we assessed the stemness of glioma cells by measuring a panel of stem cell marker genes using western blotting analysis and observed significantly reduced expression levels of CD133, Nestin, Nanog, and SOX-2 after curcumol treatment (Figure 2D).

### Curcumol increased the sensitivity of glioma cells to TMZ

**Figure 3:**
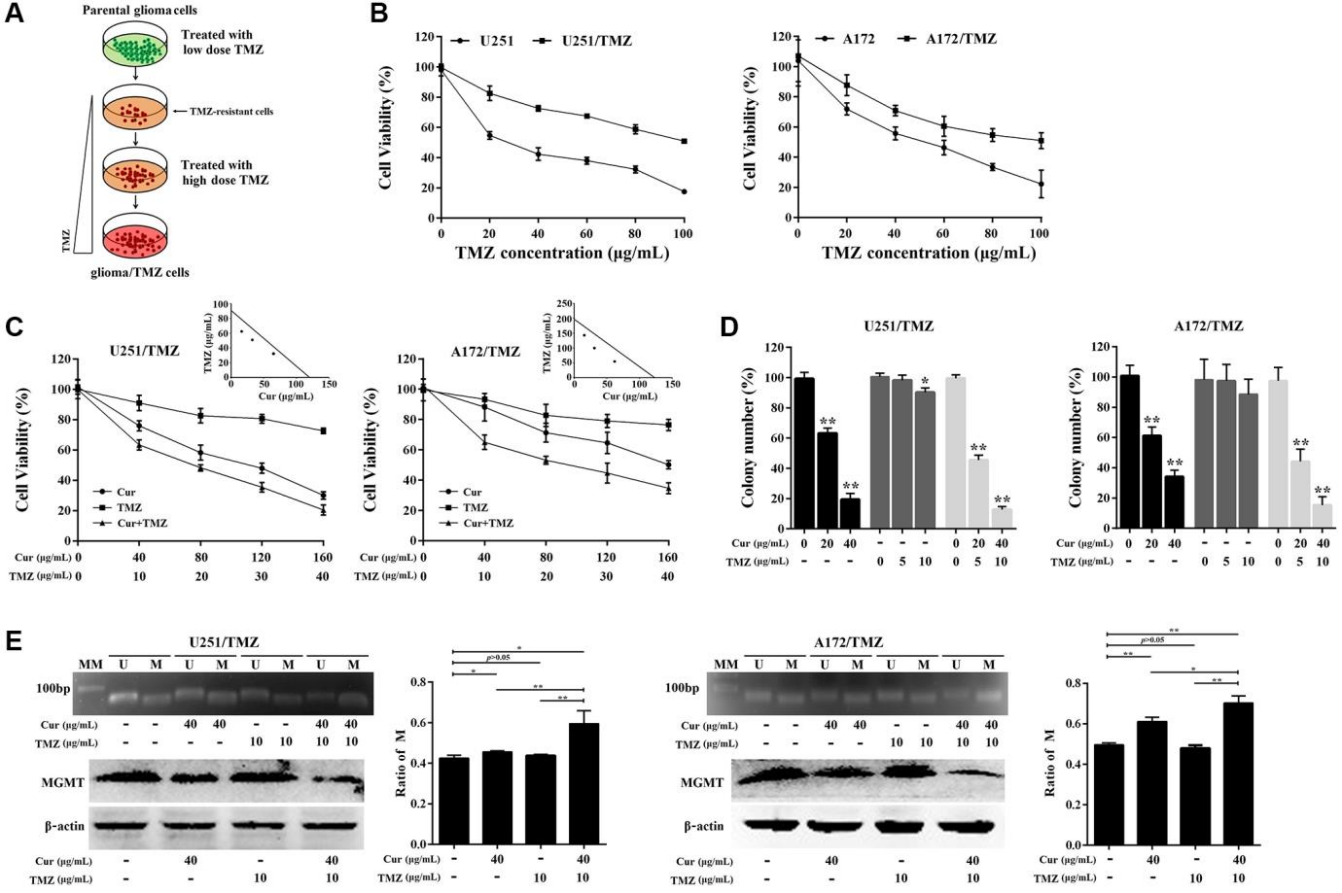
**Curcumol sensitized TMZ-resistant glioma cells to TMZ.** (**A**) Scheme for establishment of TMZ-resistant glioma cells. Briefly, the parental U251/A172 cells were cultured in DMEM medium containing increasing concentrations of TMZ until they acquired resistance to TMZ. (**B**) MTT assay was performed to compare cell viability against TMZ in resistant cells and their respective parental cells. (**C**) Isobologram analysis determined the effects of curcumol combined with TMZ on cell proliferation of TMZ-resistant glioma cells. (**D**) Colony formation assay was used to assess the clonogenicity of TMZ-resistant glioma cells following treatment with different concentrations of curcumol and/or TMZ for 14 days. (**E**) MS-PCR analysis of the MGMT promoter methylation status and the western blotting analysis of MGMT protein level in TMZ-resistant glioma cells following treatment with different concentrations of curcumol and/or TMZ. The density of each band was quantified using imaging analysis and the relative band density values were calculated as the ratio of methylated MGMT to that of methylated plus un-methylated MGMT. MGMT unmethylated: 92 bp; MGMT methylated: 8l bp; U: unmethylated; M: methylated; MM: molecular marker. ^*^P < 0.05, ^**^P < 0.01.

We generated chemoresistant cell lines against TMZ by culturing glioma cells in media containing increasing concentrations of TMZ, and then maintained them in media containing 2 μg/mL TMZ (Figure 3A). To confirm the acquired chemoresistance of these cells, we analyzed their viability at various TMZ concentrations relative to their parental counterparts. We found that both U251/TMZ and A172/TMZ cells were able to proliferate at higher concentrations of TMZ as opposed to their respective parental cell lines (Figure 3B). Next, we evaluated the effect of curcumol on TMZ sensitivity in U251/TMZ and A172/TMZ cells by determining the changes in cellular proliferation after treatment with curcumol or TMZ alone or curcumol and TMZ in combination. TMZ-resistant glioma cells appeared to show resistance to low-dose TMZ, but not to curcumol. However, the combined treatment showed enhanced cellular cytotoxicity (Figure 3C). Isobologram analysis indicated that TMZ and curcumol synergistically enhanced cytotoxicity against U251/TMZ cells (CI = 0.82 for 16.25 μg/mL curcumol combined with 62.38 μg/mL TMZ; CI = 0.83 for 32.50 μg/mL curcumol combined with 50.87 μg/mL TMZ; CI = 0.90 for 65 μg/mL curcumol combined with 32.23 μg/mL TMZ; Figure 3C) and A172/TMZ cells (CI = 0.85 for 15.38 μg/mL curcumol combined with 143.23 μg/mL TMZ; CI = 0.76 for 30.75 μg/mL curcumol combined with 99.73 μg/mL TMZ; CI = 0.78 for 61.50 μg/mL curcumol combined with 54.63 μg/mL TMZ; Figure 3C). Moreover, we performed a colony formation assay to determine the effects of curcumol and/or TMZ on the proliferative capacity and survival of TMZ-resistant glioma cells. As shown in Figure 3D, TMZ treatment alone did not effectively reduce the number of colonies of U251/TMZ cells at a lower dose (5 μg/mL) and slightly reduced the colony formation rate (10.21%) at a dose of 10 μg/mL, while neither 5 nor 10 μg/mL TMZ reduced the number of colonies of A172/TMZ cells. Curcumol treatment alone effectively suppressed the colony-forming capacity of both U251/TMZ and A172/TMZ cells, and the combination of the two compounds further inhibited the clonogenic capacity of these cell lines.

Recently, O6-methylguanine-DNA methyltransferase (MGMT) promoter methylation is implicated in drug resistance of TMZ against glioma [31]. Therefore, we confirmed the methylation status of the MGMT promoter in TMZ-resistant glioma cells treated with curcumol and/or TMZ using MS-PCR. The results showed that curcumol treatment alone significantly increased the ratio of promoter DNA methylation, and the combination of curcumol and TMZ further increased this ratio (Figure 3E). However, TMZ treatment alone did not induce the hypermethylation of the MGMT promoter region. Western blotting analysis showed that curcumol, alone or in combination with TMZ, suppressed the expression of MGMT (Figure 3E, bottom panel). In total, these results suggest that curcumol increases the sensitivity of glioma cells to TMZ.

### Curcumol treatment suppressed FOXD2-As1 expression

**Figure 4:**
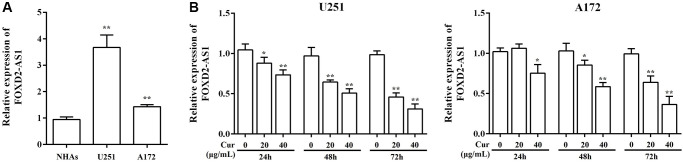
**Curcumol treatment down-regulated FOXD2-As1 expression.** (**A**) The expression of FoxD2-As1 was detected in the normal human astrocytes (NHAs) and glioma cell lines. (**B**) The expression of FoxD2-As1 was detected in glioma cells treatment with curcumol for 24, 48, and 72 h. ^*^P < 0.05, ^**^P < 0.01.

To investigate the underlying mechanism of curcumol in glioma, we explored the function of FOXD2-As1 in this process. We found that there was a marked upregulation of FOXD2-As1 expression in U251 and A172 cells compared to that in NHAs (Figure 4A). Next, we analyzed FOXD2-As1 expression in U251 and A172 cells after treatment with curcumol, for 24, 48, and 72 h. Curcumol treatment decreased FOXD2-As1 levels in glioma cells in a time- and dose- dependent manner (Figure 4B).

### FOXD2-As1 over-expression abolished the effect of curcumol on the proliferation, metastasis, and apoptosis of glioma cells

**Figure 5:**
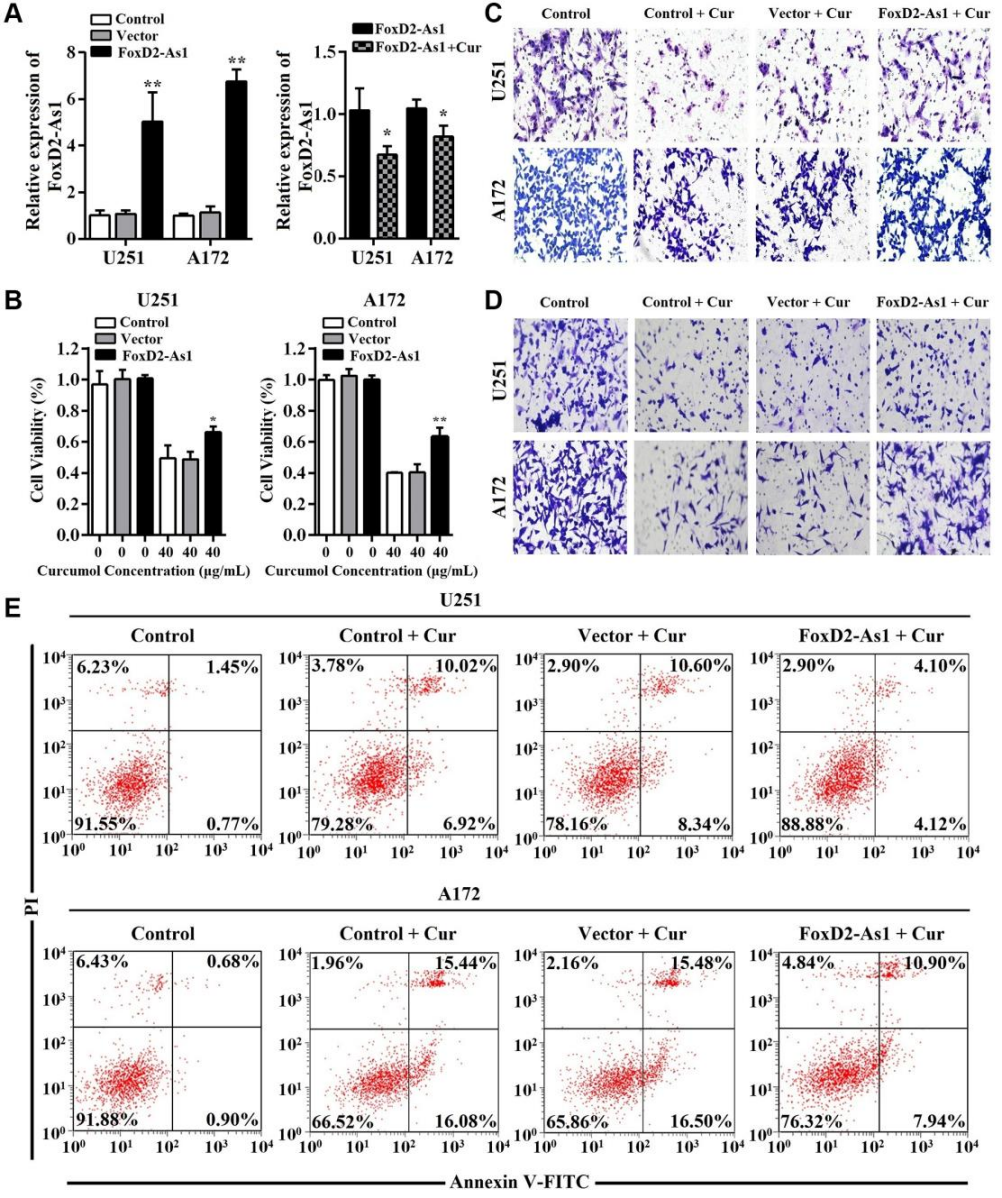
**Over-expression of FoxD2-As1 reduced the anti-proliferation, anti-metastasis, and pro-apoptosis effects of curcumol in glioma cells.** (**A**) qRT-PCR was performed to determine FOXD2-As1 expression in glioma cells of each group. pcDNA3.1-FOXD2-As1 transfection markedly increased the expression of FOXD2-As1 in glioma cells while the upregulated FOXD2-As1 expression was inhibited by curcumol treatment. (**B**) MTT assay was performed to determine the viability of each group cells after treated with curcumol for 48 h. (**C**) Transwell migration and (**D**) invasion assays were performed to examine the metastasis of each group cells after treated with curcumol for 48 h. (**E**) Annexin V-FITC/PI apoptosis assay was performed to examine the apoptotic rate of each group cells after treated with curcumol for 48 h. Data were represented as means ± SD from at least of three independent experiments. ^*^p < 0.05, ^**^p < 0.01.

Glioma cells were transfected with pcDNA3.1-FOXD2-As1 or pcDNA3.1 and then treated with curcumol for 48 h (Figure 5A). As shown through the MTT assay results, the overexpression of FOXD2-As1 remarkably promoted cell viability in curcumol-treated glioma cells compared to that in the control and pcDNA3.1-transfected cells (Figure 5B). Moreover, Transwell migration and invasion assays showed that FOXD2-As1 overexpression abrogated the curcumol-induced inhibition of cell metastasis (Figure 5C and 5D). FOXD2-As1 overexpression prominently reduced the apoptotic rate in curcumol-treated glioma cells compared to the control and pcDNA3.1-transfected cells (Figure 5E).

### FOXD2-As1 over-expression abrogated the effect of curcumol on the self-renewal ability of glioma cells

**Figure 6:**
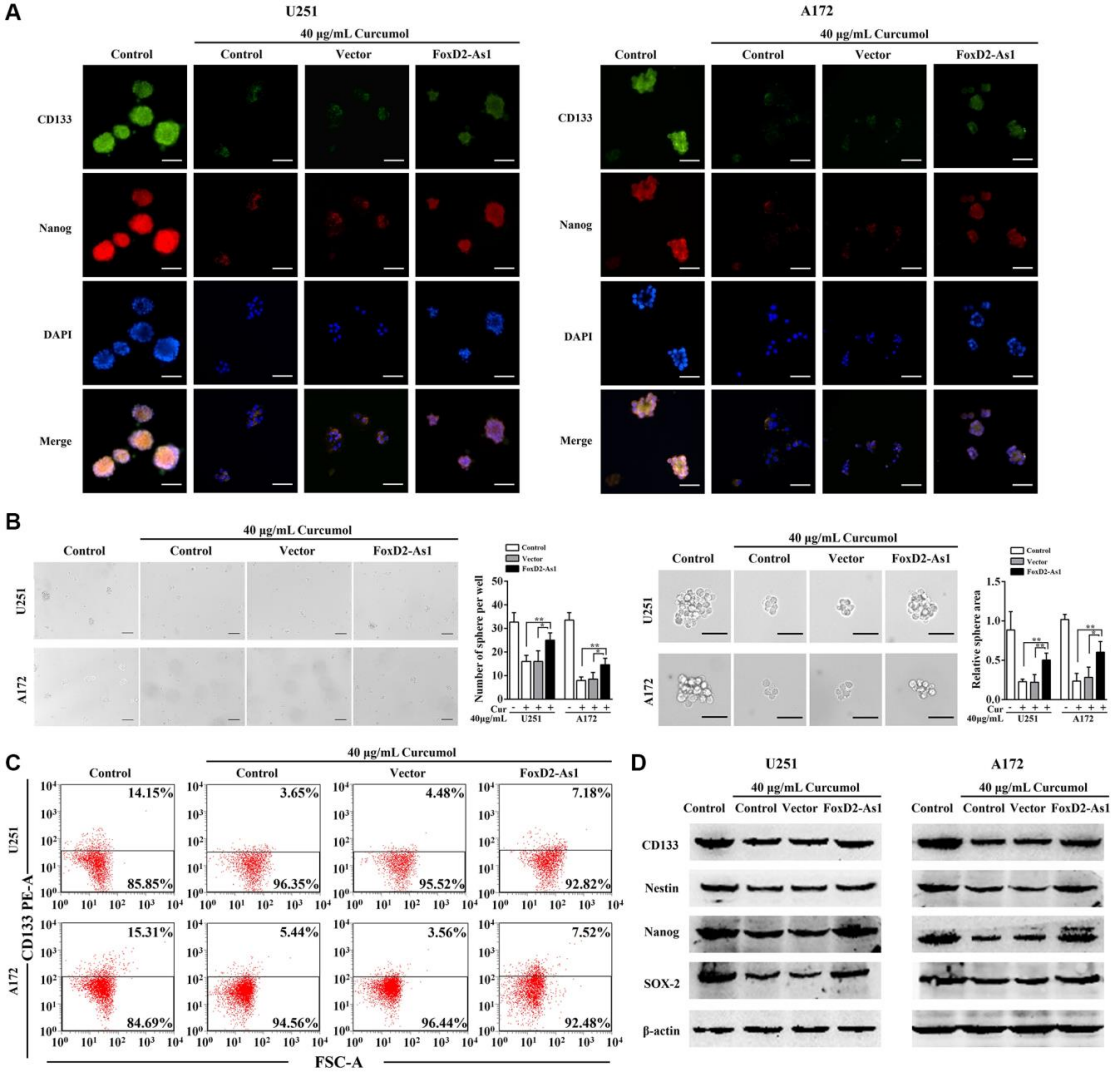
**Over-expression of FoxD2-As1 abolished the inhibitory effect of curcumol on the self-renewal ability of glioma cells.** (**A**) Immunostaining showed the expression of CD133 and Nanog on the membrane of glioma stem-like cells in each group, Scale bar = 200 μm. (**B**) Neurosphere formation assay showed that FoxD2-As1 upregulation promoted curcumol reduced neurosphere formation capacity of glioma cells in stem-like conditions, Scale bar = 150 μm. (**C**) Flow cytometry analysis showed that curcumol reduced CD133+ cells were increased by upregulation of FoxD2-As1. (**D**) Western blotting analysis showed that forced expression of FoxD2-As1 reversed curcumol-mediated decrease of CD133, Nestin, Nanog, and SOX-2 protein expression. ^*^p < 0.05, ^**^p < 0.01.

Next, we continued to determine whether FOXD2-As1 overexpression abolished the inhibitory effect of curcumol on the self-renewal ability of glioma cells. As shown in Figure 6A and 6B, FOXD2-As1 up-regulation significantly promoted neurosphere formation and expression of CD133 and Nanog in U251 and A172 cells, which grow in stem-like conditions in the presence of curcumol. The upregulation of FOXD2-As1 also increased the curcumol-mediated reduction of the CD133+ cell percentage in both U251 and A172 cell lines (Figure 6C). Moreover, western blotting analysis results showed that FOXD2-As1 up-regulation reversed the curcumol-mediated decrease in CD133, Nestin, Nanog, and SOX-2 protein expression levels (Figure 6D). These results demonstrated that the overexpression of FOXD2-As1 abolished the effect of curcumol on the self-renewal capacity of glioma cells.

### FOXD2-As1 over-expression abrogated the effect of curcumol on glioma resistant to TMZ

**Figure 7:**
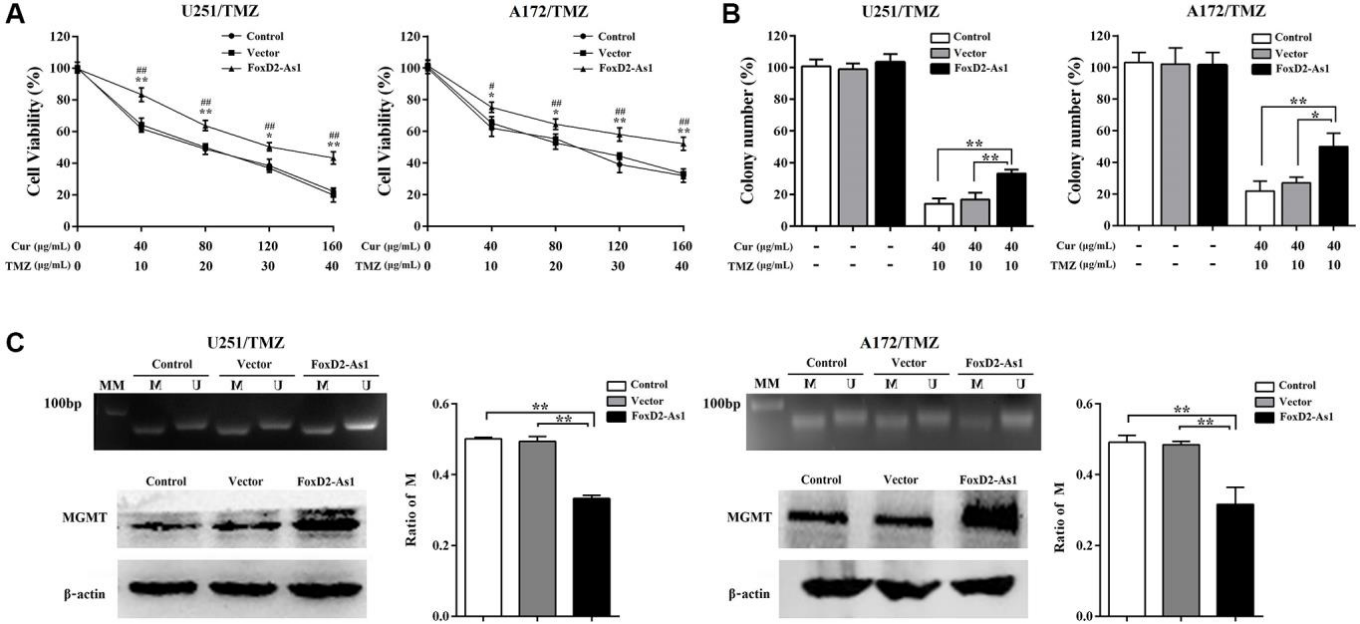
**Over-expression of FoxD2-As1 abolished the effect of curcumol on TMZ-resistance of glioma cells.** (**A**) MTT assay showed that FoxD2-As1 overexpression increased the cell viability compared to control and empty vector transfection groups. (**B**) Colony formation assay showed that the clonogenicity of TMZ-resistant glioma cells reduced by curcumol and TMZ treatment was reversed by FoxD2-As1 overexpression. (**C**) FoxD2-As1 overexpression significantly reduced the ratio of methylated MGMT promoter DNA and increased MGMT protein level in TMZ-resistant glioma cells. ^*^P < 0.05, ^**^P < 0.01. FoxD2-As1 overexpression VS. control group; ^#^*P* < 0.05, ^##^*P* < 0.01. FoxD2-As1 overexpression VS. empty vector group.

We subsequent investigated the function of FOXD2-As1 in the effect of curcumol on TMZ resistance in glioma cells. As shown in Figure 7A and 7B, FOXD2-As1 overexpression reversed the cytotoxic effects of curcumol and TMZ on U251/TMZ and A172/TMZ cells and increased their colony numbers, which were decreased after curcumol and TMZ treatment. Furthermore, compared with those in the control and empty vector cells, FOXD2-As1 overexpression significantly reduced the ratio of methylated MGMT promoter DNA and promoted MGMT protein expression in TMZ-resistant glioma cells (Figure 7C). The above mentioned results showed that FOXD2-As1 up-regulation abolished the effect of curcumol on glioma resistant to TMZ.

### Curcumol inhibited the activation of EZH2 through the downregulation of FOXD2-As1

**Figure 8:**
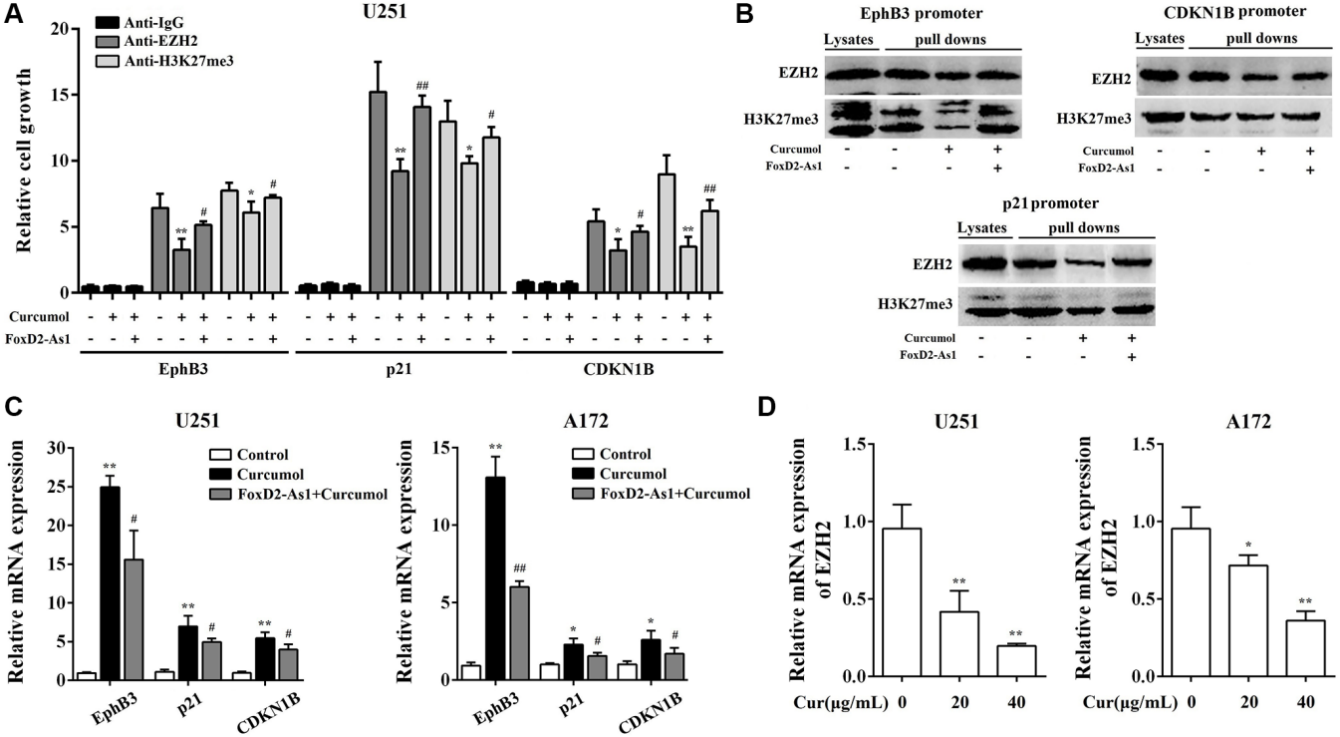
**Curcumol inhibited the activation of EZH2 through the downregulation of FoxD2-As1.** ChIP (**A**) and DNA pull down (**B**) assay showed the binding of EZH2 and H3K27me3 to the promoters of p21, EphB3, and CDKN1B. (**C**) curcumol inhibited the mRNA expression level of p21, EphB3, and CDKN1B in U251 and A172 cells. (**D**) curcumol inhibited the expression of EZH2 in U251 and A172 cells. ^*^P < 0.05, ^**^P < 0.01. FoxD2-As1 overexpression VS. control group; ^#^*P* < 0.05, ^##^*P* < 0.01. FoxD2-As1 overexpression VS. empty vector group.

Recent research has reported that FOXD2-As1 performs a tumor inducer by promoting the recruitment of EZH2 to tumor suppressor gene promoters and increasing H3K27me3 modification. We hypothesized that curcumol modulated the activation of EZH2 through FOXD2-As1 to exert antitumor activity. Thus, we first detected the activation of EZH2 in glioma cells with or without curcumol treatment. There is evidence that FOXD2-As1 epigenetically silences *EphB3* [26], *CDKN1B* [27], and *p21* [28] through EZH2; therefore, DNA pull-down and Chip assays were performed to determine how curcumol affect the recruitment of EZH2 to *EphB3*, *CDKN1B*, and *p21* promoters. As shown in Figure 8A and 8B, 40 μg/mL curcumol treatment decreased the binding ability of EZH2 to *EphB3*, *p21*, and *CDKN1B* promoters in glioma cells. Meanwhile, curcumol inhibited H3K27me3 modification in the promoter regions of *EphB3*, *p21*, and *CDKN1B*. As expected, curcumol significantly increased the levels of EphB3, p21, and CDKN1B in glioma cells (Figure 8C). These results suggest that curcumol inhibited the activation of EZH2.

To evaluate whether FOXD2-As1 is involved in curcumol-reduced EZH2 activity, we overexpressed FOXD2-As1 in glioma cells. As shown in Figure 8A and 8B, the overexpression of FOXD2-AS1 significantly obviated the inhibitory effect of curcumol on the binding ability of EZH2 and H3K27me3 modification in the promoter regions of *EphB3*, *p21*, and *CDKN1B*. FOXD2-As1 overexpression obviated the promoting effect of curcumol on the levels of EphB3, p21, and CDKN1B (Figure 8C). Moreover, we found that curcumol inhibited the expression of EZH2 (Figure 8D).

### Curcumol inhibited malignant biological behaviors and TMZ-resistance of glioma cells *in vivo*

**Figure 9:**
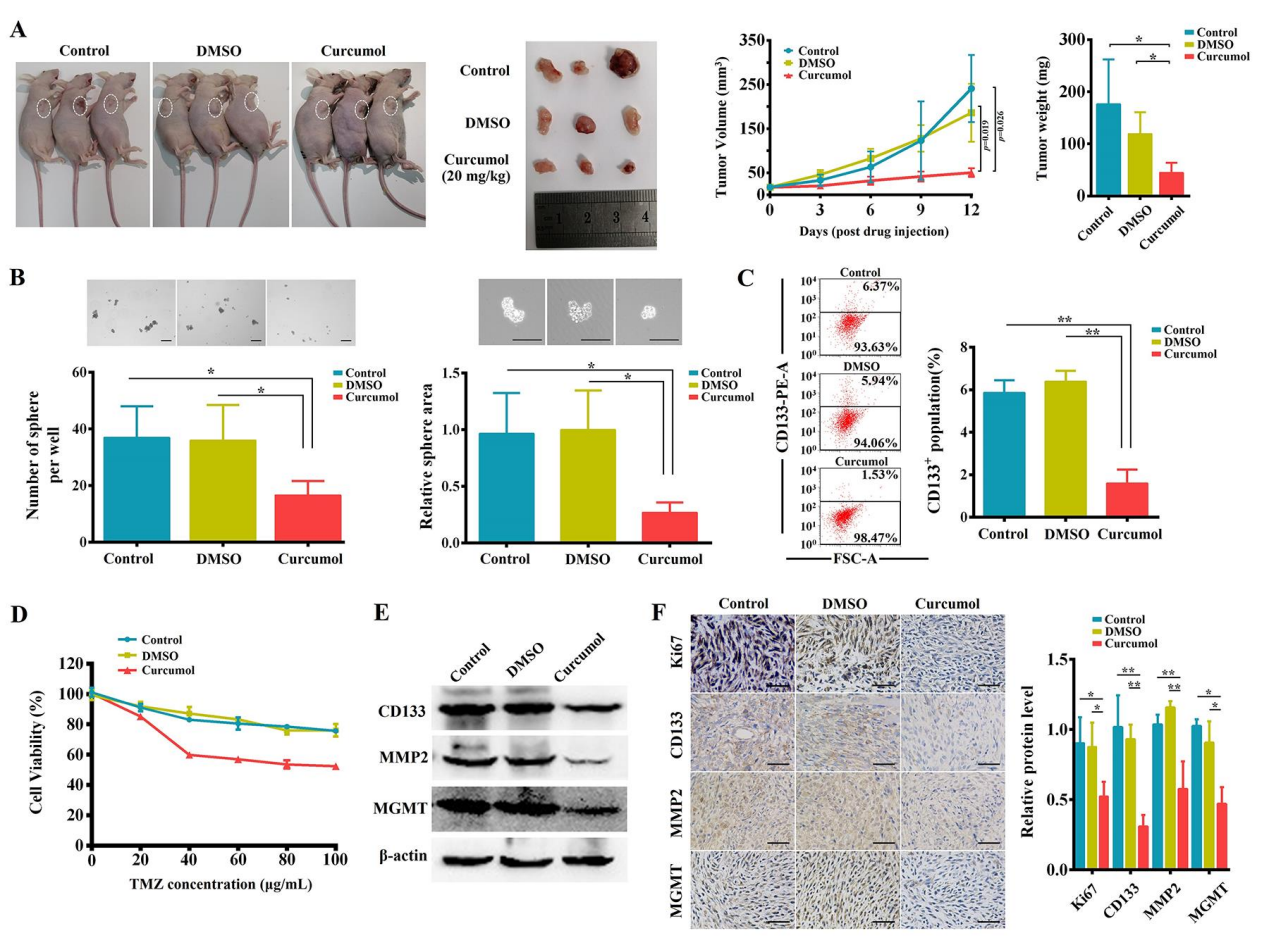
**Curcumol inhibited malignant biological behaviors and TMZ-resistance of glioma cells *in vivo*.** (**A**) Representative BALB/c nude mice and tumors from each treatment group. Curcumol treatment significantly reduced 251/TMZ cells-derived tumor volume and weight. (**B**) Tumor biopsies from curcumol group had lower ability to generate spheres as compared to those of control and DMSO groups. (**C**) The CD133+ cell percentage in tumor sample of curcumol group was obviously lower than that in control and DMSO groups. (**D**) The cells from tumor sample of curcumol group showed low tolerance to TMZ. (**E**) Western blotting results showed that curcumol reduced the level of CD133, MMP2, and MGMT in tumors. (**F**) IHC results showed that curcumol reduced the level of Ki67, CD133, MMP2, and MGMT in tumors. ^*^P < 0.05, ^**^P < 0.01.

Further, for *in vivo* studies, U251/TMZ cells were subcutaneously injected into nude mice; these mice were then intraperitoneal injected of curcumol or DMSO for 12days. Curcumol treatment significantly suppressed U251/TMZ cell-derived tumor growth as observed based upon the tumor volume and weight (Figure 9A). Then, tumor biopsies from the three groups were collected and cultured to examine the sphere-forming ability of the cells and their tolerance to TMZ. As shown in Figure 9B, under serum-deprived conditions, the tumor samples from the curcumol group showed a significantly decreased sphere-forming ability compared to that of the control and DMSO groups. Moreover, CD133+ cells in tumor samples from the curcumol group were significantly reduced compared to other groups (Figure 9C). After treatment with a series of concentrations of TMZ for 48 h, the cells from the tumor samples of the control and DMSO groups showed a high tolerance to TMZ; however, the cells from the tumor samples of the curcumol group showed a low tolerance to TMZ (Figure 9D). We then assessed CD133, MMP2, and MGMT expression in the tumors of each group using western blotting and IHC. The results showed that curcumol treatment markedly reduced the protein expression levels of CD133, MMP2, and MGMT (Figure 9E and 9F). Meanwhile, Ki-67 levels were much lower in the curcumol-treated tumors (Figure 9F). These findings demonstrated that curcumol suppressed the proliferative capacity, self-renewal capacity, metastasis, and TMZ-resistance of glioma cells *in vivo*.

## DISCUSSION

Chinese herbal medicine is an important part of complementary and alternative medicine, and its potential in the treatment of glioma has been demonstrated. In China, herbal medicine combined with radiotherapy and/or chemotherapy has achieved good results in improving the adverse reactions and the quality of life of glioma patients, preventing postoperative recurrence, and prolonging their survival [32–35]. Moreover, several preclinical studies have demonstrated an inhibitory effect of natural compounds extracted from herbal medicines on glioma cell growth [36–38]. In the present study, we focused on curcumol, a well-known herbal medicine and health food supplement in China. Our study revealed that curcumol treatment suppressed the proliferation and invasion of glioma cells, while promoting cell apoptosis. Additionally, we observed that curcumol treatment reduced the percentage of CD133+ glioma cells and stem cell markers levels in glioma cells as well as their sphere-formation ability, demonstrating the inhibitory effects of curcumol on glioma self-renewal ability.

*MGMT* promoter methylation is a key mechanism of *MGMT* gene silencing and predicts a favorable TMZ chemotherapy outcome in glioma patients. The reagent’s effect of increasing *MGMT* promoter methylation rates indicates that it may be a potential sensitizer in TMZ treatment. Here, chemoresistant cells were established to evaluate the effect of curcumol on TMZ resistance in glioma cells. Our results indicated that curcumol synergized with TMZ to reduce the viability and clonogenic capacity of TMZ-resistant cells. Synergistically with TMZ, curcumol also showed a promoting effect on the ratio of *MGMT* promoter methylation and an inhibitory effect on MGMT protein levels. In addition, curcumol treatment also showed an inhibitory effect on the growth of 251/TMZ cell xenografts. These data suggest the potential of curcumol in sensitizing glioma cells to TMZ.

The inhibitory effects of curcumol on different cancer cells involve different molecular mechanisms. In colorectal cancer cells, curcumol promoted cell death through the IGF-1R and p38 MAPK pathways [39]; in melanoma, curcumol inhibited cell proliferative ability and metastasis through the PI3K/AKT and ERK/NF-kappa B pathways [11], while in gastric adenocarcinoma cells curcumol downregulated isocitrate dehydrogenase 1 [40]. Here, we demonstrated that FOXD2-As1 might contribute to the effects of curcumol on glioma cells. An forced expression of FOXD2-As1 attenuated the curcumol-induced reduction in glioma cell proliferative capacity, metastasis, self-renewal capacity, and TMZ resistance. FOXD2-As1, located on chromosome1p33, having a transcript with a length of 2,527 nucleotides, is reported to be highly expressed in glioma tissues [29, 30]. The current study demonstrated that curcumol reduced FOXD2-As1 levels in glioma cells in a dose- and time-dependent manner, suggesting that FOXD2-As1 might be a molecular target of curcumol treatment in glioma. It is noteworthy that the repression effect of curcumol on FOXD2-As1 expression in U251 was more significant than that in A172 cells, even though FOXD2-As1expression levels were much lower in A172 cells. These results suggested that there were other genes involved in the effect of curcumol on FOXD2-As1 expression and we will further explore the interaction pattern of curcumol and FoxD2-As1 in our future work.

More recently, lncRNAs were found to act as a scaffold for various epigenetic proteins and influence the epigenetic state of chromatin to regulate tumor progression [41]. Shu et al. reported that FOXD2-As1 promoted gastric cancer carcinogenesis by EZH2 mediated epigenetically silencing EphB3 [26]. Xu et al. reported that FOXD2-As1 acts as an oncogenic gene in hepatocellular carcinoma via EZH2 mediated CDKN1B epigenetic silencing [27]. EZH2 was found to be a candidate cancer therapeutic target, with several studies suggesting its potential therapeutic benefits against glioma. We further investigated whether curcumol inhibited the malignant behavior of glioma cells by reducing FOXD2-As1-induced EZH2 activity. We found that curcumol treatment decreased the binding ability of EZH2 to the promoter of downstream effectors (*EphB3*, *p21*, and *CDKN1B*) and the H3K27me3 modification in the promoter regions of effectors and increased the expression levels of effectors in glioma. Furthermore, an forced expression of FOXD2-As1 abolished the effects of curcumol on EZH2 downstream effectors. In addition, we found that curcumol inhibited the expression of EZH2, which is consistent with the results of Li et al. [42]. Hence, we established a direct interaction between curcumol treatment and FOXD2-As1-induced EZH2 activity in glioma cells.

## CONCLUSIONS

**Figure 10:**
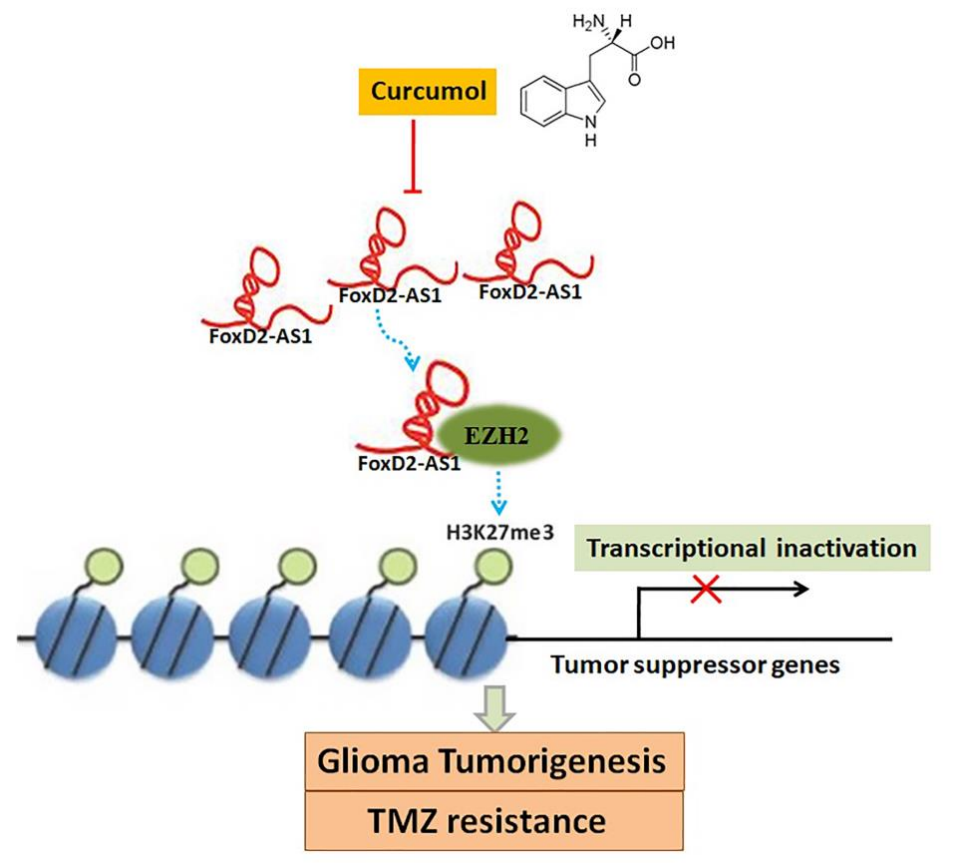
**A schema diagram displaying the inhibitory effects of curcumol on glioma tumorigenesis and TMZ-resistance.** Based on the findings of the present, FOXD2-As1 might be a molecular target of curcumol treatment, and that curcumol performs its functions in glioma cells by repressing FOXD2-As1-induced EZH2 activity.

To sum up, our data demonstrated that curcumol is effective in inhibiting the proliferative capacity, migrative and invasive ability, self-renewal capacity, and TMZ resistance of glioma cells *in vitro* and *in vivo*. Moreover, our findings showed that curcumol inhibits FOXD2-As1 expression. We believe that FOXD2-As1 might be a molecular target of curcumol, and that curcumol performs its functions in glioma cells by repressing FOXD2-As1-induced EZH2 activity (Figure 10). These results highlight the potential of curcumol as a promising therapeutic agent for glioma and may provide an option for the clinical application of natural herbal medicine. However, further investigations, including determination of a therapeutic dose of curcumol that can eliminate glioma cells while minimizing damage to normal cells, must be conducted.

## METHODS

### Cell lines and materials

Normal human astrocytes (NHAs) and glioma cell lines U251, A172 were purchased from Cell Resource Center of Shanghai and maintained in DMEM containing 10% fetal calf serum (FBS), 1% penicillin, 1% streptomycin at 37°C in 5% CO_2_. Curcumol and temozolomide (TMZ, HY-N0104 and HY-17364, MCE, USA) were dissolved in DMSO.

### RNA isolation and qRT-PCR analysis

TRIzol reagent was used to isolate total RNA from the cells and tissues. Subsequently, cDNA synthetization and qRT-PCR were performed by using HiFi-MMLV cDNA Kit and UltraSYBR Mixture (Comwei Biotech, China). The primers were synthesized by GenScript Co. Ltd. (Nanjing, China) and the sequences were illustrated as follows: FOXD2-As1, F: 5′-CTCACATCCGGCGGCT-3′, R: 5′-GGCTGTTCATGATATGTGCCA-3′;CDKN1B, F: 5′-GCCGCAACCAATGGATCTCCTC-3′, R: 5′-AGTCGCAGAGCCGTGAGCAA-3′; EPHB3, F: 5′-TGGGTAACATCTGAGTTGGCG-3′, R: 5′-TGGTATGTGCGGATGGGATTC-3′; p21, F: 5′-TGTGATGAAAGACGGCACAC-3′, R: 5′-CTTCCTTTGGGTATTGTTTGG-3′; EZH2, F: 5′-GGACCACAGTGTTACCAGCAT-3′, R: 5′-GTGGGGTCTTTATCCGCTCAG-3′; β-actin, F: 5′-GGCACCACACCTTCTACAAT-3′, R: 5′-GTGGTGGTGAAGCTGTAGCC-3′. The 2^−ΔΔCt^ method was used to calculate the fold-change of each gene.

### Genome DNA extraction and methylation-specific (MS) PCR

For the DNA extraction, Takara MiniBEST Universal Genomic DNA Kit was used, following the manufacturer’s directions. After bisulfite treatment by a DNA Methylation Kit (Comwei Biotech, China), the purified DNA was used to perform MS-PCR. Primers for the methylated MGMT were F: 5′-TTTCGACGTTCGTAGGTTTTCGC-3′, R: 5′-GCACTCTTCCGAAAACGAAACG-3′; for the un-methylated MGMT were F: 5′-TTTGTGTTTTGATGTTTGTAGGTTTTTGT-3′, R: 5′-AACTCCACACTCTTCCAAAAACAAAACA-3′. The PCR products were separated by 2.5% agarose gels and the density of each band was measured using the enhanced chemiluminescence system.

### Cell viability

Glioma cells (5 × 10^3^/well) were plated in 96-well plates. After treatment with curcumol for 48 hours, MTT solution (5 mg/mL) was added to the medium and the plates was incubated for another 4 h at 37°C. Then, the medium was removed and formazan crystals were dissolved in DMSO. The OD_550_ was detected using a SpectraMaxM3 microplate reader.

### Migration and invasion assays

Cells were suspended in DMEM and then plated into the upper chambers of the transwell inserts (Corning, USA). Afterwards, the DMEM/20% FBS was added to the lower chambers. Except for the pretreatment of the membrane with matrigel (BD Biosciences, USA), the invasion assay used the same method as the migration assay. Cells were fixed, stained and counted under ECLIPSE Ti-S microscope.

### Colony formation assay

Cells (500/well) were placed in 6-well plates and then cultured at 37°C for 2 weeks. The colonies were stained with 5% crystal violet and photographed under the microscope.

### Annexin V-FITC/PI apoptosis assay

The treated and untreated cells were collected and resuspended in the binding buffer for 15 min in dark. Then, the cells were stained with Annexin V-FITC and PI (KeyGen Biotech, China) and the apoptotic rates were detected using Guava easyCyte 5 Flow Cytometer.

### Neurosphere formation

Cells (3 × 10^4^/well) were plated into ultra-low-attached 6-well plate (Corning, USA) and suspended in serum-free DMEM/F12 medium (Gibco, USA) with 2% B27 supplement (Thermo Fisher Scientific, USA), 20 ng/mL EGF, and 10 ng/mL bFGF (PeproTech, USA). The cells were cultured for 2 weeks and fresh medium was added every 4–5 days. The spheres with diameter ≥50 μm were photographed and counted.

### Immunofluorescence staining

The neurospheres were fixed with 4% paraformaldehyde and incubated with anti-CD133 (18470-1-AP, Proteintechgruop, USA) and anti-Nanog (ab62734, Abcam, USA) overnight at 4°C. Then, SABC (rabbit IgG)-FITC/SABC (mouse IgG)-Cy3 Kit (Boster Bioengineering, China) was used. The cell nuclei were counter stained with DAPI for 10 min. Coverslips were examined by ECLIPSE Ti-S fluorescent microscope.

### Flow cytometric analysis (FCM)

Glioma cells were cultured in stem cell medium (50% DMEM, 50% F12 medium plus 2% B27, 2 mM L-glutamine, 2 μg/mL heparin, 20 ng/mL EGF, and 20 ng/mL bFGF) to establish glioma stem-like cells. One month later, cells (1 × 10^6^/well) were collected and incubated in CD133-PE antibody solution (Invitrogen, USA) for 15 min. Guava easyCyte 5 Flow Cytometer was used to analyze the percentage of CD133+ cells.

### Western blotting

Tissues and cells were lysed with RIPA containing 1 mM PMSF (Boster Bioengineering, China). Western blotting was performed based on a previous study [31]. Anti-β-actin was obtained from (Hua Bio Biotechnology, China) anti-EphB3 was obtained from (Invitrogen, USA), the antibodies to MGMT, Nestin, SOX2, p21, CDKN1B were obtained from (Proteintech Group, USA), and the antibodies to EZH2 and Histone H3 (H3K27me3) were obtained from (Abcam, Cambridge, USA).

### Isobologram analysis

The equation: combination index (CI) = (*d1/Dx1*) + (*d2/Dx2*) was used for combination assay [43]. *Dx1* is the TMZ concentration needed to produce x percentage effect alone, *d1* is the TMZ concentration needed to produce the same x percentage effect combined with *d2*. *Dx2* is the curcumol concentration needed to produce x percentage effect alone, and *d2* is the curcumol concentration needed to produce the same x percentage effect combined with *d1*. CI <1 stand for synergism, CI =1 stand for additive, and CI >1 stand for antagonism.

### Plasmids’ construction and stable transfection

The FOXD2-As1 sequence was synthesized and cloned into the pcDNA3.0 vector (GenScript, China). For establishing the FoxD2-As1 stable overexpression glioma cell lines, pcDNA-FoxD2-As1 or empty vector was transfected into cells using Lipofectamine 2000 (Invitrogen, USA). G418 was used to select the transfected cells and the transfection effect was tested by qRT-PCR.

### DNA pull down assay

Biotinylated DNA probes corresponding to the EphB3, p21, and CDKN1B promoter was prepared with PCR using glioma cell genome DNA as the template. The oligonucleotides used, corresponding to the EphB3 promoter was F: 5′-ACCCGCAGGTACTACAGTCT-3′, R: 5′-CACCGCGATGTATCCTGTGA-3′; to the p21 promoter was 5′-GAGGTCAGCTGCGTTAGAGG-3′, R: 5′-TGCAGAGGATGGATTGTTCA-3′; to the CDKN1B promoter was 5′-AAGTGCCGCGTCTACTCCTG-3′, R: 5′-TGGAGGCAGGGCAATGGT-3′. The biotinylated DNA probe (1 μg) 30 μL streptavidin agarose beads (Sigma-Aldrich) were mixed and incubated in buffer at 4°C overnight. Nuclear extracts were prepared from glioma cells using the Nuclear Extraction Kit (Takara, Japan). 400 μg of cell nuclear protein extract was incubated with DNA-coupled magnetic beads at 4°C for 1 h. After washed with cell lysis buffer and PBS, the beads were boiled for 5 min in 2 × SDS-loading buffers. Then, the samples were analyzed by western blotting using EZH2 and H3K27me3 antibodies.

### Chromatin immunoprecipitation (Chip) assay

Glioma cells were harvested after fixing with formaldehyde, and chromatin was enzymatically sheared. Subsequently, the chromatin immune precipitates were isolated on protein G magnetic beads (Active Motif, USA) using 2 μg of IgG, EZH2, or H3K27me3 antibodies. After washing, the Chips were eluted, reverse cross-linked. Then, the samples were analyzed by qRT-PCR.

### Mouse tumor xenografts

Institutional Animal Care and Use Committee of Zhejiang Chinese medical university laboratory animal research center approved all studies in this paper. BALB/c nude mice (16–20 g, Shanghai Slack Laboratory Animal Co. Ltd) were subcutaneously injected with 251/TMZ cells (1 × 10^7^/mouse). After 7 days, the mice were randomly divided into three groups (*n* = 5), and DMSO or curcumol (20 mg/kg) were intraperitoneal injected every 3 days. The animals were sacrificed and segregated the tumors for subsequent experiments.

### Immunohistochemistry (IHC)

The tumors sections were mounted on the glass slides. The specimens were treated with 3% hydrogen peroxide, blocked with 3% BSA in 0.01 M PBS, and then incubated with antibodies for Ki-67, CD133, MMP2 and MGMT. After incubation with secondary antibodies, the cell nuclei were counterstained with hematoxylin.

### Statistical analysis

Data were presented as mean ± SD. Student’s *t* test or one-way ANOVA followed by Turkey’s post hoc test was performed for the statistical analyses. *P* < 0.05 was considered statistically significant.

### Availability of data and materials

The datasets used and analyzed during the current study are available from the corresponding author on reasonable request.

### Ethics approval and consent to participate

The animal procedures were authorized by the Institutional Animal Care and Use Committee of Zhejiang Chinese medical university laboratory animal research center in compliance with the NIH Guide for Care and Use of Laboratory Animals.
